# Burnout among mental healthcare providers in Jordan: a cross-sectional study

**DOI:** 10.3389/fmed.2026.1842228

**Published:** 2026-06-10

**Authors:** Shorouq Alqannas, Hana Taha, Ahmad Shaker Abuabed, Sondos Alhawamdeh, Rania Ali Albsoul, Mishkah BaniMustafa, Ayman M. Wahbeh, Munir Abu-Helalah, Linus Jönsson

**Affiliations:** 1 Department of Family and Community Medicine, Faculty of Medicine, The University of Jordan, Amman, Jordan; 2 Department of Neurobiology, Care Sciences and Society, Karolinska Institute, Solna, Sweden; 3 Community and Mental Health Nursing, Faculty of Nursing, Zarqa University, Zarqa, Jordan; 4 Department of Obstetrics and Gynecology, Doctor Sulaiman Alhabib Medical Group, Riyadh, Saudi Arabia; 5 Department of Internal Medicine, School of Medicine, The University of Jordan, Amman, Jordan; 6 Public Health Institute, University of Jordan, Amman, Jordan

**Keywords:** burnout, depersonalization, emotional exhaustion, Maslach burnout inventory, mental healthcare providers, personal accomplishment, workload

## Abstract

**Background:**

Mental healthcare providers (MHPs) face high burnout due to emotional demands and workload pressures. In Jordan, limited resources and public stigma may further increase occupational strain, underscoring the need to assess burnout among MHPs. This study aims to assess burnout levels among MHPs in Jordan, compare burnout dimensions across professional groups, and examine associations with demographic and work-related factors.

**Methods:**

This cross-sectional study included 221 MHPs in Jordan. Burnout was measured using the Arabic Maslach Burnout Inventory (MBI), which assesses emotional exhaustion (EE), depersonalization (DP), and personal accomplishment (PA). Data was collected from February 11 to 25, 2025, through online questionnaires (Google Forms; *n* = 100) and paper-based surveys (*n* = 121). Analyses were conducted using SPSS version 29.0. Group differences were examined using t-tests and ANOVA/MANOVA, with Scheffé post-hoc tests as appropriate. Statistical significance was set at *p* < 0.05.

**Results:**

The mean scores indicated high EE (19.06 ± 12.26), low DP (6.21 ± 6.90), and moderate PA (31.57 ± 11.61). In total, 42.5% reported high EE, 71.9% low DP, and 48.4% high PA, suggesting lower burnout in this area. MANOVA identified significant effects of age (*p* = 0.036) and weekly working hours (*p* = 0.007) on burnout. Follow-up analyses found significant differences in PA by age (*p* = 0.034) and working hours (*p* = 0.003). Participants aged 45 or older reported higher PA than younger groups (Scheffé, *p* < 0.05). Those working 40–49 or 50 or more hours per week reported higher PA than those working 20–29 h per week.

**Conclusion:**

MHPs in Jordan face high EE, low DP, and moderate PA. This necessitates improving organizational support, adequate staffing, managing workloads, regulating overtime, implementing supportive supervision and well-being programs to sustain providers’ performance, enhance resilience and coping with work-related stress.

## 1 Introduction

Mental health care is highly demanding for healthcare professionals, who are frequently required to provide therapeutic interventions for clients with complex mental health issues (1). This role necessitates empathy and careful attention to patients’ experiences with loss, grief, anxiety, and depression. If not managed effectively, these demands can lead to EE and stress, which, if unaddressed, may escalate into burnout (2).

Burnout among mental healthcare providers (MHPs) is an important concern that can affect both staff well-being and the quality of patient care (1, 3, 4). Maslach and Jackson’s framework describes burnout as a three-dimensional syndrome that develops in response to ongoing workplace stress. It includes EE, which reflects feeling emotionally drained and overextended; DP, which involves becoming more distant or less emotionally engaged with care recipients; and reduced PA, which reflects lower feelings of competence and achievement at work (5, 6). This framework also highlights that burnout often reflects a mismatch between the person and their work environment, where job demands and organizational conditions can play an important role in shaping burnout experiences (7). These pressures are particularly relevant in healthcare settings, where work is emotionally demanding and workloads and working hours may be high (3).

In Jordan, MHPs encounter context-specific challenges that may intensify occupational stress and elevate the risk of burnout (8). Mental health services are limited compared to physical health services, and the mental health workforce is insufficient to meet the needs of the population (9). The WHO Mental Health Atlas 2020 (Jordan) reports a total mental health workforce of 417 professionals (4.13 per 100,000 population), including 105 psychiatrists (1.04 per 100,000) and 300 mental health nurses (2.97 per 100,000) (9). Within this context, burnout and lower perceived social support have been documented among Jordanian mental health nurses (8). Additionally, mental health stigma affecting healthcare providers may further intensify occupational strain (10). Collectively, these factors may increase the risk of burnout among MHPs (8, 10).

Therefore, this study aimed to: (1) examine the prevalence and severity of burnout among mental healthcare providers in Jordan, including psychiatrists, psychiatric nurses, and psychologists; (2) compare burnout dimensions among psychiatrists, psychiatric nurses, and psychologists; (3) assess the impact of demographic and work-related factors, such as age and weekly working hours, on burnout dimensions.

## 2 Methods

### 2.1 Study design

A descriptive cross-sectional design was utilized to investigate burnout and its associated factors among mental health care providers in Jordan. This design facilitated the collection of data from a large sample at a single time point to assess burnout levels and related variables. The study was reported in accordance with the Strengthening the Reporting of Observational Studies in Epidemiology (STROBE) guidelines (11) (Supplementary material 1).

### 2.2 Study setting

Data were collected from public-sector mental health services operating under the Jordan Ministry of Health. These services included three inpatient facilities managed by the National Center for Mental Health (NCMH): the National Center for Mental Health, the National Addiction Rehabilitation Center, and Al-Karamah Hospital (12). Additionally, four community mental health centers that provide outpatient services, as well as Al-Istishariyah Clinic (affiliated with NCMH), were included. A network of psychiatrists affiliated with NCMH provides coverage for 52 outpatient mental health clinics nationwide, including health centers, prisons, and care homes (12), which facilitated recruitment across diverse public-sector outpatient settings. Although the study initially aimed to include private and NGO facilities, participation from these sectors was restricted due to delayed approvals and clinic closures. Therefore, the final sample was predominantly obtained from public-sector facilities.

### 2.3 Participants

#### 2.3.1 Eligibility criteria

Mental health care providers were eligible to participate if they met the following criteria: (1) current employment as a psychiatrist, psychiatric nurse, or psychologist; (2) a minimum of 1 year of professional experience; and (3) current provision of direct mental healthcare services during the study period. Providers were excluded if they: (1) had employment outside the target professional groups; (2) lacked direct clinical care provision; (3) had less than 1 year of professional experience; and (4) had questionnaires with substantial missing data.

#### 2.3.2 Participant recruitment and selection

A stratified recruitment strategy was implemented to achieve proportional representation from the three target professional groups. Prior to data collection, all potential participants received study information through departmental meetings, informational posters in staff areas, and direct communication with unit managers. To accommodate varying work schedules and levels of digital access, a dual-mode data collection approach was employed. The first mode involved an online survey distributed via a QR code and a direct link to a Google Form, shared through professional networks and staff communication platforms, resulting in 100 responses. The second mode utilized a paper-based questionnaire for staff with limited online access or a preference for a physical format. To prevent duplication and promote equal participation, paper questionnaires were distributed via shift-tracking logs, allowing the research team to approach providers across shifts without reapproaching the same individual. A total of 121 completed paper questionnaires were collected. In both data collection modes, the questionnaire was accompanied by a participant information sheet that outlined the study’s purpose, voluntary participation, and confidentiality measures. Completion and return of the questionnaire, whether online or in person, constituted implied consent. The researcher was available to address procedural questions but did not interpret or clarify questionnaire items to minimize potential bias.

### 2.4 Variables

Demographic and occupational variables included sex, age group, educational level, job title, years of experience, weekly working hours, institution type, and marital status. Outcome variables included the burnout dimensions, measured as continuous scores for EE, DP, and PA, and categorized into low, moderate, and high levels according to the Arabic version guidelines (13).

### 2.5 Data sources/measurement

Data collection was conducted using a two-part questionnaire. The first section obtained sociodemographic and occupational data, including sex, age group, educational attainment, job title, years of professional experience, average weekly working hours, type of institution (inpatient or outpatient), and marital status. The second section evaluated burnout using the validated Arabic version of the Maslach Burnout Inventory–Human Services Survey (MBI-HSS) (13). The instrument was used under a formal commercial license obtained from Mind Garden, Inc. The Arabic version employed in this study was based on a previously validated adaptation (13), selected due to its demonstrated psychometric adequacy in an Arab population. This approach is consistent with licensing requirements and established research practices. This 22-item instrument comprises three subscales: EE (9 items), DP (5 items), and PA (8 items). Respondents rated each item on a 7-point Likert scale ranging from 0 (“Never”) to 6 (“Every day”). Subscale scores were summed and classified as low, moderate, or high burnout according to the established cut-off scores for the Arabic version (13). The MBI-HSS was chosen due to its established reliability and validity in Jordan, with previous studies reporting subscale-to-total score correlations of 0.76 (EE), 0.56 (DP), and 0.68 (PA), as well as a stratified alpha coefficient of 0.71 (13). Both online and paper-based formats maintained identical content, item order, response options, and instructions to ensure consistency. Domain scores were computed and interpreted according to the scoring guidelines for the Arabic version, as outlined in Table 1.

**Table 1:** Comparison of MBI scoring standards between MBI-HSS manual (3rd ed.) and Arabic versions.

Section	The Arabic version scale	The original (MBI) scale
Emotional exhaustion	The total score is 15 or less: low level of burnout	The total score is 17 or less: low level of burnout
	A total score between 16 and 18: moderate level of burnout	A total score between 18 and 29: moderate level of burnout
	A total score above 19 indicates a high level of burnout.	A total score above 30 is a high level of burnout.
Depersonalization	Total score of 8 or less: low level of burnout	Total score of 5 or less: low level of burnout
	Total score between 9 and 22: moderate level of burnout.	A total score between 6 and 11: moderate level of burnout.
	A total score above 23: high level of burnout	A total score above 12: high level of burnout
Personal accomplishment	The total score is 14 or less: high level of burnout	The total score is 33 or less: high level of burnout
	Total score between 15 and 34: moderate level of burnout.	Total score between 34 and 39: moderate level of burnout.
	A total score above 35 is a low level of burnout.	A total score above 40 is a low level of burnout.

Source: Authors’ compilation from (6, 13).

### 2.6 Bias

A stratified recruitment approach invited all eligible providers from specific groups to minimize selection bias. To address coverage bias, both online and print methods were used, with identical questionnaires provided to reduce information bias. Researchers offered procedural clarification but did not interpret questions. Duplicate responses were prevented by limiting online submissions to one per email and using shift-tracking logs for paper surveys.

### 2.7 Study size

The sample size was estimated using an online sample size calculator (Calculator.net) (14). The calculation assumed a 95% confidence level (*α* = 0.05), 80% power (*β* = 0.20), and a moderate effect size, resulting in a minimum required sample of approximately 200 participants. To account for missing data, attrition, and incomplete questionnaires, 10% was added, yielding a target sample of 220; ultimately, 221 valid responses were included in the final analysis. Comparable cross-sectional studies in mental health settings have reported similar sample sizes, including a study of Jordanian mental health nurses (*N* = 181) (8) and a study of psychiatric hospital nurses in South Africa (*N* = 198) (15).

### 2.8 Statistical analysis

Data was cleaned and coded in Microsoft Excel. Responses not to meet the inclusion criteria and questionnaires with substantial missing data were excluded. Descriptive statistics summarized the study variables: categorical variables were presented as frequencies and percentages, and burnout dimension scores (EE, DP, and PA) were presented as means and standard deviations. Burnout scores were also categorized into low, moderate, and high levels, with frequencies and percentages reported. Multivariate analysis of variance (MANOVA) was used to assess the effects of demographic and work-related factors on the scores for the three burnout dimensions. MANOVA was selected because the three burnout dimensions (EE, DP, and PA) are theoretically and empirically correlated. Joint analysis of these dimensions reduces the risk of Type I errors. Independent variables included sex, age group, educational level, job title, years of experience, weekly working hours, and marital status. When multivariate effects were significant, univariate ANOVA was conducted for each burnout dimension, followed by Scheffé *post hoc* tests for pairwise comparisons. Analyses were conducted using IBM SPSS Statistics version 29.0 (16), with statistical significance set at *p* < 0.05.

### 2.9 Ethical considerations

This study was conducted in accordance with the ethical principles outlined in the Declaration of Helsinki (17) Ethical approval was obtained from the Institutional Review Board at the Ministry of Health of Jordan (IRB No. MOH/REC/2024/20). Participation was voluntary and anonymous, and informed consent was obtained from all respondents prior to data collection. Confidentiality was strictly maintained, and all information was used only for research purposes.

## 3 Results

### 3.1 Participant characteristics

Of the 240 mental healthcare providers approached at public-sector facilities in Jordan, 221 completed the questionnaire, yielding a 92.1% response rate. 19 providers declined to participate due to time constraints or lack of interest.

### 3.2 Sociodemographic and occupational characteristics

The study included 221 mental healthcare providers: 113 males (51.1%) and 108 females (48.9%). Nearly half were aged 35 to 44 years (109; 49.3%), and most held a bachelor’s degree (172; 77.8%). Psychiatric nurses were the largest group (153; 69.2%), followed by psychiatrists (49; 22.2%) and psychologists (19; 8.6%). Seventy-one participants (32.1%) had over 16 years of experience, and most worked 40 to 49 h per week (114; 51.6%). The majority were employed in the public sector (213; 96.4%), and most were married (168; 76.0%). No data was missing for any variables of interest (Table 2).

**Table 2:** Participant characteristics (*N* = 221).

Variable	Category	(*N*)	(%)
Sex	Male	113	51.1
	Female	108	48.9
Age group	25–34 years	79	35.7
	35–44 years	109	49.3
	45 years or older	33	14.9
Educational level	Diploma	25	11.3
	Bachelor’s degree	172	77.8
	Postgraduate studies	24	10.9
Job title	Psychiatrist	49	22.2
	Psychiatric nurse	153	69.2
	Psychologist	19	8.6
Years of experience	1–5 years	68	30.8
	6–10 years	45	20.4
	11–15 years	37	16.7
	More than 16 years	71	32.1
Working hours per week	Less than 20 h	8	3.6
	20–29 h	15	6.8
	30–39 h	57	25.8
	40–49 h	114	51.6
	50 h or more	27	12.2
Type of institution	Public sector	213	96.4
	Non-governmental organization (ngo)	5	2.3
	Private sector	3	1.4
Marital status	Single	43	19.5
	Married	168	76.0
	Widowed/divorced	10	4.5

### 3.3 Burnout levels

The mean scores for burnout dimensions were 19.06 (SD 12.26) for EE, 6.21 (SD 6.90) for DP, and 31.57 (SD 11.61) for PA. Based on Arabic MBI-HSS cut-off scores, 94 participants (42.5%) exhibited high levels of EE. The majority of participants (159; 71.9%) demonstrated low DP. Regarding PA, 107 participants (48.4%) were classified in the low-burnout category (high PA), whereas 20 participants (9.0%) were categorized in the high-burnout group (low PA) (Table 3).

**Table 3:** Burnout levels among Jordanian mental health providers.

Burnout subscale	Mean (SD)	Low burnout *n* (%)	Moderate burnout *n* (%)	High burnout *n* (%)
Emotional Exhaustion (EE)*	19.06 (12.26)	107 (48.4%)	20 (9.0%)	94 (42.5%)
Depersonalization (DP)**	6.21 (6.90)	159 (71.9%)	53 (24.0%)	9 (4.1%)
Personal Accomplishment (PA)***	31.57 (11.61)	107 (48.4%)	94 (42.5%)	20 (9.0%)

*EE, emotional exhaustion (low ≤15, moderate 16–18, high ≥19).

**DP, depersonalization (low ≤8, moderate 9–22, high ≥23).

***PA, personal accomplishment (low ≥35, moderate 34–15, high ≤14).

### 3.4 Factors associated with burnout

A one-way MANOVA assessed the relationships between demographic and occupational variables and the three dimensions of burnout. The analysis found statistically significant effects for age group (Wilks’ *Λ* = 0.935, *F* = 2.268, *p* = 0.036) and weekly working hours (Wilks’ Λ = 0.875, *F* = 2.310, *p* = 0.007) on the combined dependent variables. No significant effects were found for sex (*p* = 0.543), education level (*p* = 0.715), job title (*p* = 0.328), years of experience (*p* = 0.415), or marital status (*p* = 0.326) (Table 4).

**Table 4:** MANOVA analyses of the association between demographic/work-related factors and burnout dimensions (*N* = 221).

Factor	Multivariate test	Value	*F*	*p*-value^*^
Sex	Hotelling’s Trace	0.011	0.716	0.543
Age group	Wilks’ Lambda	0.935	2.268	0.036
Education level	Wilks’ Lambda	0.982	0.619	0.715
Job title	Wilks’ Lambda	0.966	1.157	0.328
Years of experience	Wilks’ Lambda	0.956	1.030	0.415
Working hours/week	Wilks’ Lambda	0.875	2.310	0.007
Marital status	Wilks’ Lambda	0.982	1.161	0.326

^*^p < 0.05 indicates statistical significance.

Following the significant multivariate results, univariate ANOVAs were conducted as follow-up analyses. These showed that the significant effects were limited to the PA dimension. PA scores differed significantly by age group (*F* = 3.437, *p* = 0.034) and weekly working hours (*F* = 4.211, *p* = 0.003) (Table 5).

**Table 5:** Follow-up ANOVA analyses of the association between demographic/work-related factors and burnout dimensions (*N* = 221) (significant findings only).

Burnout dimension	Factor	df	*F*	*p*-value^*^
Personal accomplishment	Age group	2	3.437	0.034
Personal accomplishment	Working hours/week	4	4.211	0.003

^*^p < 0.05 indicates statistical significance.

Scheffé *post hoc* comparisons showed that participants aged 45 years or older reported significantly higher PA than those aged 25–34 years (mean difference = −5.77, *p* = 0.04) and 35–44 years (mean difference = −5.96, *p* = 0.03). For working hours, individuals working 40–49 h per week (mean difference = −10.25, *p* = 0.024) and 50 h or more per week (mean difference = −11.42, *p* = 0.038) reported significantly higher PA than those working 20–29 h per week (Table 6).

**Table 6:** Scheffé post-hoc comparisons Analyses of the association between demographic/work-related factors and burnout dimensions (*N* = 221) (significant findings only).

Outcome	Comparison (I vs J)	Mean difference (I − J)	Std. error	*p*-value^*^
Personal accomplishment	25–34 vs. ≥ 45	−5.7656	2.28	0.040
Personal accomplishment	35–44 vs. ≥ 45	−5.9611	2.19	0.030
Personal accomplishment	20–29 h vs. 40–49 h	−10.2526	3.02	0.024
Personal accomplishment	20–29 h vs. ≥ 50 h	−11.4222	3.55	0.038

^*^p < 0.05 indicates statistical significance.

## 4 Discussion

This cross-sectional study assessed burnout and related factors among MHPs in Jordan (8). Results showed high EE, low DP, and moderate PA (1, 4). Multivariate analysis found that only age and weekly working hours were significantly associated with burnout, especially PA. Providers aged 45 or older and those working at least 40 h per week reported higher PA than younger providers and those with fewer hours (8).

### 4.1 Prevalence and patterns of burnout among MHP

The observed pattern of high EE, low DP, and moderate PA in this sample supports the conceptualization of burnout as a multidimensional syndrome (2–4). The predominance of EE is consistent with international research, which identifies emotional exhaustion as the most significant component of burnout in healthcare, particularly within mental health services (2–4). Systematic reviews further indicate a high prevalence of burnout among mental health professionals, with EE frequently identified as the primary concern (1, 18, 19).

The high level of EE in the sample likely reflects ongoing challenges in Jordan’s mental health services. Most respondents work in public-sector settings, which face significant staffing and resource shortages (9, 12, 20). This distribution may be explained by the study setting, since the data were collected from three inpatient facilities and one outpatient mental health center. The inpatient facilities require continuous 24-h nursing coverage. In addition, mental health nurses represent the largest segment of the mental health workforce in Jordan (300 out of 417 professionals; approximately 71.9%) (9). Taken together, these factors suggest that the observed sample composition reflects both the mental health workforce structure and the characteristics of the study setting. The WHO has reported continued workforce shortages and service gaps in Jordan’s mental health system (9, 20). Recent analyses highlight systemic barriers, including fragmented governance, workforce turnover, and a focus on training without adequate follow-up or supervision (3). These issues contribute to sustained workload pressures and limited organizational support, leading to emotional fatigue among providers (1, 18, 19, 21, 22).

### 4.2 Factors associated with burnout

The multivariate analysis indicated that only age and weekly working hours were significantly associated with burnout dimensions. Sex, educational level, job title, years of experience, and marital status did not demonstrate a significant effect. These findings are consistent with existing literature, which reports that organizational and workload-related factors exert a greater influence on burnout than demographic characteristics (1, 3, 18). Furthermore, a recent rapid review of reviews on health workforce well-being emphasized the necessity for systemic changes and organizational accountability to protect mental health, rather than focusing exclusively on individual characteristics (9).

However, the impact of working hours is complex and requires careful interpretation. While longer weekly hours were significantly associated with higher PA, EE also tended to increase with longer hours, though this was not statistically significant. The lack of significant predictors for EE, despite its high prevalence (42.5%), may be attributed to sample homogeneity (96.4% public sector) and a potential ceiling effect. Participants may have been exposed to similar systemic stressors, resulting in limited variability in EE scores across groups, which could reduce the ability to detect significant associations. Thus, longer working hours do not necessarily reduce burnout. The findings indicate that greater work involvement and responsibility may enhance PA but also increase emotional demands. Consistent with previous research (23), job involvement may mediate the relationship between EE and job satisfaction, with longer hours and greater professional commitment sustaining a sense of accomplishment. This aligns with qualitative evidence that professional meaning and commitment can coexist with exhaustion under sustained workloads (24, 25), and with research showing that PA can remain stable even when emotional strain is high (1, 18).

### 4.3 Depersonalization and therapeutic engagement

In contrast to EE, DP levels were low in this study, and no demographic or occupational variables were significantly associated with DP. These results suggest that Jordanian MHPs maintain therapeutic engagement with patients despite emotional strain. In contrast, studies in other settings have found higher DP among mental healthcare providers (22, 26), indicating that DP may vary by context and system, possibly due to differences in workplace conditions and support. The collectivist cultural values in Jordan, which emphasize family-centered care and interpersonal connection (15), may help protect against cynicism and detachment.

Low DP scores may reflect Jordan’s collectivist cultural values, which emphasize group harmony and interpersonal connection. Social desirability bias and mental health stigma may also lead providers to underreport cynicism or detachment, as these attitudes are considered unprofessional. Collectivist values can also protect against DP by fostering group belonging and shared responsibility. In these contexts, structured peer support, family-inclusive practices, and team-based decision-making can reinforce social cohesion and reduce emotional isolation and individual burden. Evidence shows that social support and team cohesion help mitigate burnout among mental healthcare workers (8, 27), indicating that these strategies may be effective in similar cultural settings.

### 4.4 Personal accomplishment: age and experience

PA showed moderate overall levels and was most strongly linked to work-related factors in this analysis. PA varied significantly by age and weekly working hours. Participants aged 45 or older reported higher PA than younger colleagues. Those working 40–49 or at least 50 h per week also reported higher PA than those working 20–29 h per week. These findings are consistent with evidence that greater professional experience increases confidence, coping ability, and perceived competence among MHPs (1, 18, 21).

The predominance of participants aged 35–44 likely reflects the structure of the public mental health workforce in Jordan, where many professionals remain in mid-career roles. However, this pattern may also be influenced by the study design, which may have limited the inclusion of early-career staff. As age can affect burnout, this should be considered when interpreting the findings.

The link between age and higher PA likely reflects the development of clinical expertise. The finding that older (≥45 years) and more experienced participants reported higher PA, despite longer working hours, may reflect a potential survivor bias. This suggests that individuals who remain in the profession tend to be more resilient, while those who experiencing early burnout are more likely to leave (28). Previous research indicates that resilience tends to increase with age and experience among healthcare workers (27), which may contribute to more effective coping strategies and a stronger sense of professional identity. Accordingly, this finding should be interpreted with caution, as it may reflect the characteristics of a more experienced workforce rather than a direct protective effect of workload. More experienced practitioners often have stronger emotional regulation, realistic expectations, and a deeper understanding of therapeutic processes (24, 25). This view aligns with research showing that professional experience improves resilience and perceived efficacy in mental health work (1, 18). The presence of higher PA alongside elevated, though non-significant, EE in those with longer working hours highlights that PA may remain stable even under emotional strain. These results underscore the need for institutional strategies that reduce emotional demands and support professional efficacy (20, 24).

### 4.5 Systemic and organizational implications

The findings reveal both vulnerability and resilience among Jordan’s mental health workforce. EE was high, while DP remained low, and PA was moderate for most providers. This pattern underscores the need for targeted institutional strategies in public mental health services, including workload regulation, optimized staffing, supportive supervision, and staff well-being initiatives. While clinical supervision was not directly measured in this study, existing evidence highlights its important role in reducing burnout among healthcare professionals. In the Jordanian context, national reports indicate gaps in structured supervision, suggesting that strengthening supervisory systems may represent a relevant strategy to support workforce well-being (8, 9). A bibliometric analysis confirms that organizational factors are consistently identified as key antecedents across occupations (29), which supports the emphasis on systemic interventions. These measures are critical to reducing emotional fatigue and maintaining professional motivation and quality of care (24, 30). Recent policy developments, such as the National Mental Health and Substance Use Action Plan 2022–2026 (12) and the 2025 Staff Care and Self-Care policy, reflect growing recognition of these needs. The establishment of a Staff Care Center and the training of core trainers, who have conducted over 230 activities, further support this progress. Evidence shows that organizational interventions are more effective than individual approaches in addressing burnout (31). The WHO Special Initiative for Mental Health has also prioritized systemic strengthening, workforce development, and service integration (30). Additional progress includes the region’s first mental health investment case and expanded training through the Mental Health Gap Action Program (mhGAP). Despite these advances, challenges remain in ensuring sustained follow-up, supervision, and outcome evaluation (20). The recent integration of psychosocial support principles into health centers and hospitals, as highlighted by Jordan’s MOH (12), demonstrates a commitment to holistic care that addresses both physical and mental health. This comprehensive approach is essential for reducing emotional fatigue and supporting professional motivation and quality of care (24, 30).

### 4.6 Limitations

Several limitations should be considered when interpreting the study findings. **First**, the cross-sectional design measures burnout at a single point in time, so it cannot establish causality. Longitudinal studies with repeated measures are needed to track burnout over time. **Second**, the 14-day data collection window may not capture potential seasonal fluctuations. However, the chronic nature of burnout and the sample’s experience level mitigate this concern. **Third**, including only participants with at least 1 year of experience may have excluded newly graduated professionals, who are often more vulnerable to early-career burnout. Fourth, reliance on self-reported questionnaires may introduce information bias, including social desirability and recall bias. **Fifth**, financial strain or workload intensity, both predictors of burnout, were not directly measured. Working hours served as a proxy for occupational demands. **Sixth**, although recruitment targeted diverse settings, most participants were from public-sector facilities due to access constraints and clinic closures in the private and non-governmental sectors, which may limit the generalizability of the findings. **Seventh**, data collection was especially challenging in some facilities, such as the Addiction Rehabilitation Center, due to access restrictions and reliance on administrators for survey distribution. **Eighth**, the exclusion of providers from Jordan University Hospital, due to a lack of ethical approval, also limits representation from academic medical settings. Despite these challenges, the study exceeded the *a priori* sample size target (*N* = 221) and provides valuable baseline data on burnout among mental health professionals in Jordan’s public sector.

#### 4.6.1 Implications of the study

These findings have important implications for policy, practice, and organizational development. The high levels of emotional exhaustion among Jordanian mental health professionals indicate a need for systemic interventions to regulate workload, ensure adequate staffing, and manage overtime. Since PA is linked to age and working hours, interventions should address both emotional strain and professional efficacy through mentoring, skills development, and structured feedback.

At the policy level, the MOH should prioritize staff well-being in national mental health plans by supporting adequate staffing, monitoring overtime and shift schedules, and promoting fair workload distribution. Including staff well-being indicators in quality assurance frameworks may improve patient outcomes and support service sustainability. Future research should use longitudinal, multi-site studies, including the private and non-governmental sectors, to investigate causal pathways and evaluate the effectiveness of organizational and individual interventions. Future studies should also include work-environment variables such as caseload, shift patterns, and perceived organizational support to better explain burnout in the Jordanian context.

## 5 Conclusion

Burnout is a significant occupational concern among MHPs in Jordan, marked by high EE, reduced DP, and moderate PA. EE remained consistently high, while PA varied significantly by age and weekly working hours. Although EE was higher among younger professionals and those with longer working hours, these differences were not statistically significant and should be interpreted cautiously. The findings support existing evidence that workload and organizational factors are stronger predictors of burnout than demographic characteristics. Targeted interventions, such as workload optimization, enhanced supervisory support, and comprehensive well-being programs, are recommended to reduce emotional strain and maintain motivation and quality of care. Enhancing organizational support systems may further strengthen the resilience and commitment of Jordan’s mental healthcare workforce.

## Data Availability

The raw data supporting the conclusions of this article will be made available by the authors, without undue reservation.
